# Multiculturalism, Culture Mixing, and Prejudice: Effects of Priming Chinese Diversity Models Among Hong Kong University Students

**DOI:** 10.3389/fpsyg.2021.691858

**Published:** 2021-07-23

**Authors:** Frank Tian-Fang Ye, Emma E. Buchtel

**Affiliations:** ^1^Centre for Special Educational Needs and Inclusive Education, The Education University of Hong Kong, Hong Kong, China; ^2^Department of Psychology, The Education University of Hong Kong, Hong Kong, China

**Keywords:** social distance, culture mixing, cultural identity, multiculturalism, assimilation

## Abstract

In two studies, we investigated how Hong Kong university students reacted to descriptions of China as multicultural vs. assimilatory, examining effects on emotions, prejudice toward Mainland Chinese, attitudes toward Hong Kong/China culture mixing, and cultural identities. Study 1 compared a multicultural priming condition to a control condition and found that the multiculturalism prime significantly reduced desire to socially distance from Mainland Chinese. Study 2 compared multiculturalism, assimilation, or control primes’ effects, and found that the multiculturalism prime, through increased positive emotions, indirectly reduced social distancing from Mainland Chinese and disgust toward culture mixing, and increased Chinese ethnic identity and multicultural identity styles; the assimilation prime had the opposite indirect effects through increasing negative emotions. Results show new evidence of the importance of emotion in how non-immigrant regional groups, who are both minority and majority culture members, react to different diversity models. Multicultural frames increased positive emotions, with downstream positive effects on both intergroup attitudes and integrated identities.

## Introduction

Since its 1997 handover from Britain to the People’s Republic of China, Hong Kong’s has had increasing integration and interactions with Mainland China. Unfortunately, as Hong Kong becomes more integrated into the economic and political systems of Mainland China, hostility and bias toward Mainland Chinese in the city have also grown (Hong et al., 2006; Lam et al., 2006; Chou, 2012; Ma, 2015; Ng et al., 2017). Negative local attitudes are closely associated with political events, which can also create challenges for locals in navigating the implications of having a Chinese identity (Fung, 2004); for example, public opinion polls have documented an increasing preference for the “Hongkonger” identity relative to a “Chinese” identity over the last decade (The University of Hong Kong, 2019). The psychological impact of such cultural experience has gained growing attention from researchers in the field of Hong Kong identity studies (Brewer, 1999; Hong et al., 2004; Lam et al., 2006; Cao et al., 2014).

Different from past research on immigrant minorities who are navigating integration into a majority culture, Hong Kong represents a relatively unusual (though not unique) social situation where a region as a whole is experiencing integration. Little is known about whether residents’ emotional responses and intergroup attitudes can be improved by different depictions of such integration. Focusing on Hong Kong local residents, the main objective of the current research is to evaluate how different social models describing how Hong Kong is part of China—either focusing on multiculturalism or on assimilation— can affect attitudes and feelings related to Mainland China. We find that reading about different social models, mediated through emotional reactions to them, can affect Hong Kong locals’ desire to socially distance from Mainland Chinese people, disgust toward cultural mixing with Mainland China, and adoption of integrative multicultural identity management strategies.

### Models of Cultural Diversity

Multiculturalism emphasizes the appreciation of different cultural values, traditions, and lifestyles, in contrast to color-blindness or assimilation, which reflect a preference toward ignoring cultural differences (Morris et al., 2015). Research, especially in European contexts, has found that minorities, more than majority group members, tend to appreciate multicultural ideologies of society. For example, Brug and Verkuyten (2007) examined how minorities in the Netherlands viewed four types of societal models of intercultural contact: mosaic (or multiculturalism; maintaining cultural differences but working and living together), assimilation (minority groups abandon their own cultures to become part of the majority culture), melting-pot (cultural fusion between both majority and minority cultures), and segregation (groups stay apart from one another). Consistent with previous findings by Verkuyten (2005), Turkish minority group members endorsed multiculturalism, while eschewing both assimilation and separation. Among minorities, multiculturalism endorsement or exposure is also associated with positive attitudes toward the self and one’s ingroup. For example, endorsement of multiculturalism was associated with stronger ingroup identification and more positive evaluations of the ingroup (Verkuyten, 2005; Brug and Verkuyten, 2007), and higher self-esteem for high ethnic-group identifiers (Verkuyten, 2009). Experimental studies also found that exposure to multicultural depictions of society was associated with more positive self- and ingroup-evaluations, especially when compared with assimilation framing (Verkuyten, 2005, 2009). Overall, these studies suggest that immigrant minorities have more positive attitudes and reactions to multicultural recognition than assimilation or color-blind ideologies.

#### Effects on Intergroup Attitudes?

But how do these ideologies affect attitudes toward outgroups? While majority group members’ endorsement of multiculturalism is often associated with more positive attitudes toward minorities, the effects of experimental priming, and on minorities’ attitudes toward outgroups, are less clear. Among Mainland Chinese (not living in Hong Kong), multiculturalism endorsement was found to buffer against the negative effects of perceived value incongruence on attitudes toward Hong Kong Chinese (Guan et al., 2011), but among White Americans, the effects of priming multiculturalism on intergroup bias are only sometimes positive, depending on whether it is perceived as a threat to identity (Richeson and Nussbaum, 2004; Morrison et al., 2010). In the Netherlands, Verkuyten (2005, 2011) found that majority group members who endorsed or were exposed to multicultural ideologies were more likely to hold positive attitudes toward minority groups, and vice-versa for assimilation. But turning to the case of minorities, Turkish minority group members’ endorsement of multiculturalism was simply not correlated with outgroup evaluations, and minorities experimentally exposed to multicultural ideologies, vs. assimilation, even tended to have less positive evaluations of majority-culture outgroups (Verkuyten, 2005). On the other hand, among minorities in Mauritius, the endorsement of multiculturalism was associated with less social distancing from outgroups, though only for relatively public situations (e.g., willingness to work with outgroups) and not close romantic relations (Ng Tseung-Wong and Verkuyten, 2014). Thus, how and why minorities’ intergroup attitudes are affected by multicultural vs. assimilatory framing is still an important question.

In the Hong Kong context, appreciation of multiculturalism is promoted in local values education, aiming to improve ethnic and racial tolerance within Hong Kong (Au, 2010; Curriculum Development Council HKSAR, 2014). Among Hong Kong locals, endorsing multiculturalism within Hong Kong has been found to be associated with more contact with Mainland Chinese immigrants (Hui et al., 2015), similar to findings for European majority culture members in the above studies. But while locally born, Chinese ethnicity Hong Kong residents are a cultural majority within Hong Kong, they may consider themselves to be cultural minorities within China. As such, like minorities in other cultures, they may be likely to have more positive reactions to multicultural, vs. assimilatory, depictions of China as a whole and Hong Kong’s place in the country. But do such depictions lead to having more positive China-related attitudes, such as toward having a Chinese identity, toward interacting with Mainland Chinese people, or toward cultural mixing with Mainland China? This is the main question to be addressed in the following studies.

### Cultural Identities in the Hong Kong Context

The cultural situation of Hong Kong within China is different compared to typical European and Western-context immigrant societies. For example, Hong Kong residents’ interpretations of their own identity has unique features. Chinese-ethnicity Hong Kong participants may identify with superordinate identities such as being Chinese national citizens (“中國人”), and/or as being Chinese “people” in an ethnic or cultural sense (“中華民族”) (Chen et al., 2019). Individuals may also differ in whether they consider their Hong Kong and Chinese identities as integrated or separate (Fung, 2004; Ward et al., 2018; The University of Hong Kong, 2019; Szabó et al., 2020).

In the years leading up to Hong Kong’s 1997 return to China, the number of residents claiming a “Hong Kong” identity increased, potentially as a way to meet “optimal distinctiveness” needs for both belonging and differentiation (Brewer, 1999). Instead of feeling a need to differentiate themselves from Western culture, for some residents the focus shifted to developing an identity that emphasized distinctiveness from the rest of China (Brewer, 1999; Lam et al., 1999; Lau et al., 2003; Chiu and Hong, 2003). Brewer (1999) suggested that those who emphasized a Hong Kong identity would be highly sensitive to signals that China valued Hong Kong’s distinctive features (e.g., its “special contribution to China’s economy and culture;” p. 196).

Integrated identities can be especially fragile in times of social change. For example, among Hong Kong adults, perceived speed of social change was associated with a feeling of uncertainty, which was in turn associated with a preference for only one single identity rather than a dual Hong Kong and Chinese one (Kim and Ng, 2008), though most interviewed residents (62.8%) claimed a dual identity at that time. Integrated Hong Kong-Chinese identities could also be a sign of more positive attitudes toward being a part of China. Hong Kong Chinese who self-categorized into identities that prioritized their Chinese identity showed more positive attitudes toward Mainland Chinese (Hong et al., 2004, 2006; Lam et al., 2006), and the fall in residents who identify as Chinese or integrated identities such as “Hong Kong Chinese” is commonly seen as a sign of growing anti-Mainland China sentiment (The University of Hong Kong, 2019). It is thus of interest to study whether being presented with assimilatory vs. multicultural models of China would be more likely to move Hong Kong locals toward superordinate Chinese identities and/or integrated Chinese-Hong Kong identities.

### Social Distancing From Mainland Chinese

Desire to keep one’s social distance from outgroups (Bogardus, 1925, 1947), ranging from being unwilling to marry an outgroup member up to even being unwilling to live in the same city, is a significant indicator of prejudice in intergroup relations (Wark and Galliher, 2007; Parrillo and Donoghue, 2013). In Hong Kong, Chan and Goto (2003) found that Hong Kong participants preferred more social distance from Mainland Chinese than from Americans, and more desire for social distancing predicted more willingness to engage in confrontational procedures for solving work disputes. Notably, less social distancing from outgroups is associated with integrated identities; for example, among Chinese New Zealanders, a hybrid Chinese-New Zealander identity predicted more positive outgroup evaluations of other ethnic minorities (Ng Tseung-Wong et al., 2019).

### Disgust Toward Culture Mixing

In our globalized world, culture mixing is an everyday phenomenon, commonly depicted in logos and international brands that show different cultural symbols coexisting together, or even combining and fusing together (Chiu et al., 2009; Hao et al., 2016). Still, negative reactions toward culture mixing are commonly found; connecting culture mixing to sensitivity to physical contamination, several studies have found that even for symbolic representations, fused cultural icons are rated as more disgusting compared to the standalone original icons (Cheon et al., 2016). Negative responses to culturally fused images are proposed to be a form of cultural defensiveness, exaggerated when the mixing is perceived as invasive (Cui et al., 2016; Yang et al., 2016) or by local identification; for example, greater identification with Singapore or Hong Kong was related to disgust toward fused local-non-local images (Cheon et al., 2016; Cheon and Hong, 2020), and in Hong Kong, local identification also predicted increased disgust toward any images involving Mainland Chinese elements (Cheon and Hong, 2020).

### Emotions as Mediators

Finally, especially in the current emotionally charged context of Hong Kong’s continued social and political integration into Mainland China, participants’ affective response to reading about different diversity models of China could importantly guide their effects on intergroup attitudes and identities. As mentioned above, Brewer (1999) emphasized for those who hoped China would highly value Hong Kong’s distinctive features, perceptions that these expectations were not being met could lead to anger and resistance.

Emotional reactions to different models of social diversity have been understudied, but research has shown that emotions have important effects on intergroup processes, and understanding them is vital to both understanding and improving intergroup relations (Mackie et al., 2008; Maitner et al., 2017). Among majority group members, anger and other negative emotions toward outgroups have been associated with less support of immigration policies (Verkuyten, 2004; Visintin et al., 2017; Verkuyten et al., 2018), and more group actions of minorities against majority group members (Hayward et al., 2018). Several studies have found that intergroup contact leads to more positive outgroup attitudes, mediated by increased positive emotions (e.g., empathy) and decreased negative emotions (e.g., anger and anxiety; Miller et al., 2004; Hayward et al., 2017; Seger et al., 2017). These studies emphasize the important role of emotions in intergroup processes. In a rare study of emotional reactions to multicultural vs. other frames, for majority group members, reading multicultural-framed news regarding immigrant outgroups led to reduced anger, which in turn mediated the frame’s effects on improving outgroup attitudes (Lecheler et al., 2015).

Thus, we propose that priming societal models that relate to outgroups could elicit emotional responses; specifically, especially among a young university student population, multicultural primes might lead to more positive emotions than assimilation primes, which could serve as the underlying processes that explain their effects on attitudes toward outgroups.

### The Current Research

In these studies, we examined the effects on Hong Kong residents of presenting cultural diversity within China in terms of either multiculturalism or assimilation. Study 1 first tested the direct effects of a multicultural prime (compared to no prime) on reducing social distancing toward Mainland Chinese, reducing disgust toward Hong Kong-Mainland China culture mixing, and enhancing national identity strength. Results were weak, suggesting mixed reactions to the multiculturalism prime. Thus, in Study 2, we also examined the mediating role of emotional reactions to the primes and added an assimilation prime for comparison. As will be seen below, emotions were a significant mediator of the priming effects, through which beneficial effects of multiculturalism and harmful effects of assimilation were found on social distancing, disgust toward culture mixing, Chinese ethnic identity strength and multicultural Chinese-Hong Kong identity styles.

## Study 1

In the context of political and economic integration into Mainland China, Hong Kong locals may have concerns about cultural identity, such as whether holding a local identity is compatible with holding a superordinate identity. As described above, immigrant cultural minorities tended to endorse multiculturalism in previous research (Verkuyten, 2005, 2009), and similarly, Hong Kong residents might also appreciate reading about Hong Kong being a valued part of multicultural China (a multicultural frame). We thus tested if a multicultural frame prime, compared to a control, would lead participants to have more positive reactions to Mainland China in general, in the forms of exhibiting less prejudice toward Mainland Chinese, being less disgusted by pictures that mix iconic Mainland China and Hong Kong images, and being more willing to identify with a superordinate, national Chinese identity.

In summary, we proposed the following hypotheses in Study 1:

*Hypothesis 1*. Participants primed with multiculturalism will exhibit less social distancing from Mainland Chinese compared to those under the no prime condition.

*Hypothesis 2*. Participants primed with multiculturalism will exhibit less disgust toward culture mixing compared to those under the no prime condition.

*Hypothesis 3*. Participants primed with multiculturalism will exhibit greater national identity strength compared to those under the no prime condition.

### Method

#### Participants

One hundred and eighteen^1^ undergraduate students, all of whom self-identified as Hong Kong permanent residents, were recruited from a university in Hong Kong through online advertisements sent by email and posted to university-internal portals^2^. Participants filled out an online survey. They were informed that this was a study regarding their understanding of cultures, and instructed to finish a 5-min online questionnaire consisting of the measurement tools described in the next section and demographic questions. Upon beginning the questionnaire, participants were randomly assigned into either the priming condition or the no prime condition. All items in the questionnaire were presented in Traditional Chinese. The final sample for data analysis consisted of 118 participants (87 females, 30 males and 1 unreported, *M*_*age*_ = 21.69, SD = 4.69) with 57 participants in the priming condition and 61 participants in the no prime condition. Upon completion of the study, five participants were compensated HK$50 (about United States $5) based on a lucky draw.

#### Procedure and Measures

##### Priming manipulation

Participants were randomly assigned to either the Multiculturalism Prime group or the No Prime group. The instructions and Multiculturalism priming material were adapted from previous research in the United States (Todd and Galinsky, 2012) with content modified through group discussions to fit the Hong Kong cultural context. Following the consent and information sheet, participants in the Multiculturalism Prime group read a paragraph describing the cultural diversity of the Greater China area, including Hong Kong as one of the contributing cultures, and were asked to briefly list up to five reasons to support why multiculturalism is beneficial to Greater China. A translated sample of the Multiculturalism Prime material is given below:

“There are many different cultural groups living together in the Greater China region, and this is where we are, in such a unique place … Each group in the Greater China region can make contributions in a different way, including IT in Shenzhen, the financial industry in Hong Kong, …Recognizing this diversity can help creating a harmonious relationship among different ethnic groups …”

As in Todd and Galinsky (2012), participants in the No Prime group did not read any further materials, but were offered the chance to list up to five questions regarding the study.

##### Social distancing

Social distancing from Mainland Chinese was measured using the Bogardus Social Distance Scale adopted from Miller and Salkind (2002). Participants rated agreement with 5 items on a 7-point Likert scale (1 = extremely disagree to 7 = extremely agree). Items began with the stem “I am willing to accept Mainland Chinese as…”, from “kinship by marriage” to “citizen of my country”. Item ratings were reverse-scored and averaged to form an index of social distancing. The scale yielded excellent reliability, McDonald’s ω = 0.90 and Cronbach’s α = 0.90.

##### Disgust (toward culture mixing)

Disgust toward Hong Kong-Mainland Chinese culture mixing was measured using five artificially processed images that consisted of a mixture of Hong Kong and Mainland Chinese cultural symbols, independently developed but based on the model of Cheon et al. (2016) that measured disgust toward images of Chinese-Western culture mixing (see Supplementary Materials for example images). Five pairs of typical or iconic images representing Hong Kong and Mainland China respectively were selected through group discussion with both Hong Kong and Mainland Chinese students. Then, new “mixed” images that presented a Hong Kong cultural symbol as the major portion, with the Mainland Chinese cultural symbol occupying a minor portion, were created to represent five “home culture modified by foreign culture” mixed culture stimuli. These five images were presented in random order for each participant with the question “To what extent do you feel disgust when you see this picture?” (as in Cheon et al., 2016). Responses were rated on a 6-point Likert scale from “not at all” (1) to “extremely” (6). The scale yielded good reliability; McDonald’s ω = 0.84 and Cronbach’s *α* = 0.84.

##### National identity strength

Participants’ national identity strength was measured with four group-identity items adopted from Reed and Aquino (2003), modified to measure endorsement of a national Chinese identity (“中國人”). In Chinese common terminology, this word specifically implies one is a member of China the country/nation, rather simply than being of Chinese ethnicity. Sample items include “It’s great to be [National] Chinese”, “I am extremely proud of my affiliation with [the Chinese nation]”. Participants rated the four items on a 7-point Likert scale (1 = extremely disagree to 7 = extremely agree). The scale yielded acceptable reliability, McDonald’s ω = 0.80 and Cronbach’s α = 0.74.

### Results^3^

Analyses testing the priming effects between two conditions were conducted in JASP (JASP Team, 2018) using independent sample *t*-tests (see Table 1 for bivariate correlations of DVs). The results showed that the multiculturalism (MC) priming significantly reduced social distancing compared to the control condition, *t*(116) = −2.19, *p* = 0.03, Cohen’s *d* = 0.40, 95% CI = [0.04,0.77]; *M*_*control*_ = 4.41, *SD* = 1.26; *M*_*MC*_ = 3.89, *SD* = 1.31; but had no effect on disgust, *t*(116) = −1.33, *p* = 0.188, Cohen’s *d* = −0.24, 95% CI = [−0.61,0.12]; *M*_*control*_ = 2.87, *SD* = 1.26; *M*_*MC*_ = 2.56, *SD* = 1.26; nor national identity strength, *t*(116) = 1.32, *p* = 0.187, Cohen’s *d* = 0.25, 95% CI = [−0.12,0.61]; *M*_*control*_ = 3.59, *SD* = 1.18; *M*_*MC*_ = 3.87, *SD* = 1.13. In summary, compared to a no-prime control, the multiculturalism prime significantly decreased social distancing but had no statistically significant effects on disgust toward Hong Kong-Mainland culture mixing or national identity strength.

**TABLE 1:** Study 1: Bivariate Pearson Correlations.

Variable	1	2
1. Social Distancing	–	
2. Disgust	0.48***	–
3. National Identity	−0.70***	−0.47***

Correlations calculated across all participants in Study 1 (N = 118), without controlling for condition. ***p < 0.001.

## Study 2

In Study 1, priming Hong Kong university students with a multicultural description of China (vs. a control condition) significantly reduced their desire to socially distance themselves from Mainland Chinese individuals. However, it did not demonstrate a significant effect on promoting national identity strength or reducing disgust toward images that insert Mainland Chinese iconic images into iconic Hong Kong images, i.e., culturally mixed images. The direct effect of multicultural priming compared to a control condition might be weak in Hong Kong for two reasons. While multiculturalism has been promoted in Hong Kong for years, with the aim of increasing tolerance of minorities within Hong Kong, the effects of describing China as a whole as multicultural might be mediated by students’ individual reactions, especially on measures related to identity and interregional relations. In particular, a significant decrease in the strength of identification with the “[National] Chinese” identity compared to other identities such as “Hong Konger” has been observed among Hong Kong local residents since the 1997 handover (The University of Hong Kong, 2019), and students’ national identity may be difficult to shift with priming. Similarly, reactions to the culturally mixed images may have been dependent on whether participants saw them through the lens of negative or positive feelings, e.g., of invasion vs. creative fusion.

In Study 2 we sought to address these issues by, first, measuring identity in a way that would be less likely to evoke pre-set reactions, and second, by examining mediation through emotions. First, compared to the term describing a national Chinese identity used in Study 1 (“中國人”), the term for ethnic Chinese (“中華民族”) describes the population in a broader sense, and a higher percentage of Hong Kong residents identify with it (The University of Hong Kong, 2019). Thus, it was used to test how diversity models would affect the strength of identifying with an “ethnic Chinese” identity among Hong Kong participants.

Additionally, Study 2 compared the multiculturalism and control primes with an assimilation prime that emphasized the absorption of a Hong Kong identity into a Chinese identity, in contrast to the multicultural Greater China emphasized by the multiculturalism prime. Based on aversive reactions from minorities toward assimilation attested in prior European studies (Verkuyten, 2005), the assimilation prime was hypothesized to elicit various negative effects opposite to those generated by the multiculturalism prime.

In Hong Kong, we may predict that reading about Hong Kong as a valued part of multicultural China may trigger stronger positive emotions than negative emotions, and vice versa for the assimilation priming:

*Hypothesis 1:* Assimilation-primed participants will experience stronger negative affect, whereas multiculturalism-primed participants will experience stronger positive affect, compared to each other and the control group.

Similarly, and following Study 1, the two primes are expected to have contrasting effects on prejudice-relevant measures, i.e., social distancing and disgust toward culturally mixed images:

*Hypothesis 2.* Assimilation-primed participants will desire more social distance from Mainland Chinese, and judge mixed culture stimuli as more disgusting, whereas multiculturalism-primed participants will desire less distance from Mainland Chinese, and judge mixed culture stimuli as less disgusting, compared to the control group and each other.

Parallel effects are expected to occur for cultural identities. Different from the national identity measured in study 1, in Study 2 we measured a willingness to accept Chinese identity in two ways: ethnic Chinese identity, and Multicultural Identity Styles (hybrid cultural identity style and alternating identity style) that reflect different forms of accepting a mixture of National Chinese and Hong Kong local identities. As reviewed earlier, Hong Kong residents may differ in whether they consider their Hong Kong and Chinese identities as integrated or separate (Fung, 2004; The University of Hong Kong, 2019). Those who perceive these two identities are simultaneously compatible may endorse a hybrid identity style (HIS), while those who think the two identities are both valid but should be adopted in different contexts may endorse an alternating identity style (AIS) (Ward et al., 2018). As suggested by research on the role of multicultural identity styles in the cultural integration process (Ward et al., 2018; Szabó and Ward, 2019), the multiculturalism prime could promote participant willingness to integrate, thereby increasing both a superordinate ethnic Chinese identity and different forms of mixed Hong Kong/Chinese identity, whereas negative reactions to the assimilation prime could discourage willingness to integrate, thereby reducing endorsement of such identities. Thus, we proposed:

*Hypothesis 3.* Assimilation-primed participants will rate themselves lower on ethnic Chinese identity, and multicultural identity styles, whereas multiculturalism-primed participants will rate themselves higher on both, compared to each other and the control group.

Finally, building on Hypothesis 1, we further hypothesized a mediating role of emotion for the Hypothesis 2 and 3 effects, such that emotional responses to the primes may subsequently explain the primes’ effects on intergroup attitudes and identity:

*Hypothesis 4.* Positive and negative affective responses will mediate the priming effects on social distancing, disgust toward culture mixing, ethnic identity strength, and multicultural identity styles.

The above hypotheses 1, 2, and 3 were preregistered, while Hypothesis 4, mediation by emotion, was not in the preregistration.

### Method

#### Participants

In total 168 university campus participants (35 males, 133 females; *M*_*age*_ = 20.8, SD = 2.91, range from 17 to 42), were recruited from a university in Hong Kong through online advertisements sent through the university-internal email system^4^. All of them were Hong Kong permanent residents and had not participated in previous studies of this project. The ideal sample size for the study was calculated using G^∗^power with the same effect size (*d* = 0.47) used in Study 1, which found that 180 participants would provide 80% power in one-way ANOVA to detect significant differences between the 3 independent priming groups^5^. Required power for detecting indirect effect was calculated with the same effect size (for path a and path b) using Monte Carlo simulation (Schoemann et al., 2017), which found that 186 participants were required to achieve 80% power. Given the sample size of 168 participants, this study is therefore slightly underpowered.

#### Procedure

Participants were recruited to participate in a study regarding their understanding of cultures, then instructed to visit a computer lab to complete a 25-minute questionnaire on individually designated computers. Upon entering the lab, they were greeted by a native Cantonese speaker and instructed to sit down in preassigned distantly spaced seats, to prevent them from interacting or observing others’ surveys. Participants were randomly assigned to one of three groups: assimilation prime (*N* = 54), multiculturalism prime (*N* = 58), or control (*N* = 56). After reading the corresponding priming materials, participants proceeded to complete responses for the remaining measurements as described in the following section, as well as other measures less relevant to the intergroup attitudes analyzed here. Each participant received HK$50 as compensation upon completing the questionnaire.

#### Measures

##### Priming manipulation

Priming conditions were implemented by random assignment via Qualtrics. Multiculturalism-primed participants read a paragraph describing the celebration of diverse cultural phenomena in the Greater China area; aside from some slight modifications, the priming material was identical to that used in Study 1. Assimilation-primed participants read a paragraph describing the 1997 handover of Hong Kong and the assimilation of the “Hongkonger” identity into the “Chinese national” identity. The priming material was adapted from a previous study about assimilating into superordinate identities (Stone and Crisp, 2007) and modified to emphasize the assimilation process. A translated sample of the material is given below:

“China resumed its sovereignty over Hong Kong in 1997. Some people think that since Hong Kong has returned, it is no longer meaningful to use ‘Hong Kong people’ to describe themselves. Instead, they think they should use ‘[national] Chinese’. In 2008, the proportion of Hong Kong people who identified themselves as “Chinese” increased by 7–12% compared with that of 2007 …”

Control group participants read a paragraph of neutral text of a similar length about the chemical element of iron, adapted from Wikipedia. A sample of the material is reproduced below:

“Iron is a chemical element with chemical symbol is Fe, atomic number 26, and relative atomic mass 56. It is a type of transitional metal. Iron is the most commonly used material on earth…”

After reading these materials, participants in all three groups were presented with a question item asking “to what extent do you agree with the above paragraph?” Responses were rated on a 7-point Likert scale from “strongly disagree” (1) to “strongly agree” (7) that included a “neutral” (4) score. The question item was then followed by a writing task related to the priming material: For both the assimilation and multiculturalism priming groups, participants were asked to briefly list up to five reasons for supporting the argument in the paragraph, whereas control group participants were asked to briefly list up to five reasons why iron is useful.

##### Positive emotion (positive minus negative affect/PANAS scale)

Immediately following the priming materials, the emotional state of participants was measured using a modified 10-item version of the Positive Affect and Negative Affect Schedule (PANAS) scale originally developed by Watson et al. (1988). The scale consists of two subscales, with 5 items measuring positive affect and another 5 items measuring negative affect. Responses for all items were rated on a 6-point Likert scale from “not at all” (1) to “extremely” (6). The Positive Affect scale yielded excellent reliability, McDonald’s ω = 0.91 and Cronbach’s *α* = 0.91; also the Negative Affect scale, McDonald’s ω = 0.89 and Cronbach’s *α* = 0.89. To simplify results description, a combination of positive minus negative affect, termed ‘‘Positive Emotion,’’ was calculated by subtracting the mean score of negative affect from positive affect.^6^ Positive values indicate stronger positive emotional reaction, and the negative values indicate stronger negative emotional reaction.

##### Social distancing

Social distancing from Mainland Chinese was measured using the same scale used in Study 1. The scale in the current study showed excellent reliability, McDonald’s ω = 0.92 and Cronbach’s *α* = 0.92.

##### Disgust toward culture mixing

Disgust toward culture mixing was measured by same images and disgust ratings as used in Study 1. The scale yielded good reliability in the current study, McDonald’s ω = 0.86 and Cronbach’s *α* = 0.86.

##### Strength of ethnic Chinese identity

The ethnic identity strength of participants was measured using the same modified 4-item measure (Reed and Aquino, 2003) applied in Study 1, with the identity in question being the “ethnic Chinese” (中華民族的一員) identity. Sample items included “It’s great to be ethnically Chinese” and “I am extremely proud of my affiliation with ethnic Chinese people.” The scale yielded acceptable reliability, McDonald’s ω = 0.87 and Cronbach’s *α* = 0.82.

##### Multicultural identity styles scale (MISS)

Multicultural identity styles (i.e., Hybrid Identity Style [HIS] and Alternating Identity Style [AIS]) were measured using the scale developed by Ward et al. (2018). In the current study, the two identities measured were “Hongkonger” and “Chinese national” for the Hong Kong participants. Sample HIS items included “For me, being a Hongkonger and being a Chinese national are intermingled” and sample AIS items included “I can be a Hongkonger or a Chinese national depending on the situation”. All question items were rated on a 7-point Likert scale from “strongly disagree” (1) to “strongly agree” (7) that included a “neutral” (4) score. The HIS subscale yielded excellent reliability, McDonald’s ω = 0.93 and Cronbach’s *α* = 0.93. The AIS subscale yielded acceptable reliability, McDonald’s ω = 0.78 and Cronbach’s *α* = 0.78.

### Results

Analyses testing the priming effects between three conditions were conducted in JASP (JASP Team, 2018) using ANOVA.

An overall ANOVA predicting positive emotion from the three priming conditions showed that the primes had statistically significant different impact, *F*(2,165) = 15.19, *p* < 0.001, η^2^ = 0.16. *Post hoc* analyses with Tukey correction showed that participants in the multiculturalism (MC) prime group (*M* = 1.14, *SD* = 1.38) had higher positive emotion compared to the assimilation (A) prime group (*M* = −0.16, *SD* = 1.42), *t* = 5.45, *p* < 0.001, Cohen’s *d* = 0.93, but there was no significant difference when compared to the control group (*M* = 0.69, *SD* = 0.94), *t* = 1.94, *p* = 0.13, Cohen’s *d* = 0.39; however, the assimilation prime group had lower positive emotion compared to the control group, *t* = −3.50, *p* = 0.002, Cohen’s *d* = 0.70. Thus, Hypothesis 1 was mainly supported.

However, ANOVA analyses did not find any significant priming effects on social distancing, *F*(2,165) = 1.10, *p* = 0.33, or disgust toward culture mixing, *F*(2,165) = 1.45, *p* = 0.44, *post hoc* tests found no significant differences between any two conditions (social distancing: *p*s > 0.32, *M*_*MC*_ = 3.98, *SD* = 1.44, *M*_*A*_ = 4.24, *SD* = 1.46, *M*_*control*_ = 3.86, *SD* = 1.23; disgust: *p*s > 0.45, *M*_*MC*_ = 2.69, *SD* = 1.40, *M*_*A*_ = 2.74, *SD* = 1.41, *M*_*control*_ = 2.44, *SD* = 1.11). Thus, Hypothesis 2 was not supported.

ANOVA analysis predicting Chinese ethnic identity strength from priming conditions indicated a significant main effect, *F*(2,165) = 4.06, *p* = 0.019. *Post hoc* tests with Tukey correction showed that ethnic identity strength decreased under the assimilation prime (*M* = 3.38, *SD* = 1.76) compared to both the control (*M* = 3.93, *SD* = 1.25), *t* = 2.42, *p* = 0.043, Cohen’s *d* = 0.45, and multiculturalism prime (*M* = 3.95, *SD* = 1.11), *t* = 2.53, *p* = 0.033, Cohen’s *d* = 0.49, suggesting a defensive response to the assimilation prime, but was not influenced by the multiculturalism prime compared to control, *t* = 0.09, *p* = 0.99, Cohen’s *d* = 0.02. However, ANOVA analysis found no significant priming effects on either of the multicultural identity styles (HIS or AIS), *p*s > 0.21. Thus, Hypothesis 3 was only partially supported.

Summarizing the above, we found a significant effect of priming conditions only on emotions and ethnic identity strength, but not on HIS, AIS, social distancing, or disgust toward culture mixing. However, the significant correlations between emotions and MISS, social distancing, and disgust (Table 2) implied that potential indirect effects might be masked or suppressed by other factors (MacKinnon et al., 2007).^7^ We thus continued to test for indirect effects of the primes through positive emotions.

**TABLE 2:** Study 2: Bivariate Pearson Correlations.

Variable	1	2	3	4	5
1. Positive Emotion	–				
2. Social Distancing	−0.32***	–			
3. Disgust	−0.35***	0.48***	–		
4. Ethnic Identity	0.57***	−0.53***	−0.54***	–	
5. HIS	0.43***	−0.69***	−0.52***	0.67***	–
6. AIS	0.39***	−0.53***	−0.35***	0.51***	0.78***

Correlations calculated across all participants in Study 2 (N = 168), without controlling for condition. HIS, Hybrid Identity Style; AIS, Alternating Identity Style. ***p < 0.001.

The proposed mediation models were tested in path analysis using lavaan (Rosseel, 2012) and joint significance of indirect paths were estimated by maximum likelihood estimation with robust standard errors. The priming condition variable was treated as multicategorical. To first compare the priming conditions to control, the three conditions were recoded into two dummy variables with the control group (coded 0) as a reference group and the other two conditions coded as 1 in turn (Hayes and Preacher, 2014). An alternative dummy coding with Assimilation group coded 0 and the other two conditions coded as 1 in turn then compared the effects of Multiculturalism vs. Assimilation.

Summarized in Table 3, the results comparing the priming conditions to Control showed that both priming conditions did indirectly affect all DVs (social distancing, disgust toward culture mixing, ethnic identity strength, HIS and AIS) through emotions. Compared to control, the multiculturalism prime, through increasing positive emotion, marginally reduced social distancing and significantly reduced disgust; it also significantly increased ethnic identity strength, HIS, and AIS. On the contrary, the assimilation prime, through reducing positive emotion, significantly increased social distancing and disgust; it also significantly decreased ethnic identity strength, HIS, and AIS. A *post hoc* power analysis using package “mc_power_med” (Schoemann et al., 2017) with Monte Carlo simulation method showed a moderate to high level of power for detecting these indirect effects.

**TABLE 3:** Summary of indirect effects of primes through positive emotions in study 2.

DV	*a*	*b*	Indirect	95% CI	Power
**Multiculturalism (1) vs. Control (0)**	0.16*				
Social Distancing		−0.34***	−0.05^†^	[−0.11,0.00]	0.56
Disgust		−0.40***	−0.07*	[−0.12, −0.01]	0.59
Ethnic Identity		0.58***	0.09*	[0.01,0.18]	0.53
HIS		0.50***	0.08*	[0.01,0.15]	0.56
AIS		0.44***	0.07*	[0.01,0.13]	0.56
**Assimilation (1) vs. Control (0)**	−0.29***				
Social Distancing		−0.34***	0.10**	[0.04,0.16]	0.97
Disgust		−0.40***	0.12***	[0.05,0.18]	0.98
Ethnic Identity		0.58***	−0.17***	[−0.26, −0.08]	0.97
HIS		0.50***	−0.15***	[−0.22, −0.07]	0.97
AIS		0.44***	−0.13**	[−0.21, −0.04]	0.97
**Multiculturalism (1) vs. Assimilation (0)**	0.45***				
Social Distancing		−0.34***	−0.15***	[−0.24, −0.07]	0.98
Disgust		−0.40***	−0.18***	[−0.26, −0.11]	0.99
Ethnic Identity		0.58***	0.26***	[0.16,0.37]	0.99
HIS		0.50***	0.23***	[0.14,0.32]	0.99
AIS		0.44***	0.20***	[0.11,0.30]	0.99

The multicategorical dummy codes of priming and control conditions were indicated in the brackets. Path a (dummy codes predicting Positive Emotion), path b (Positive Emotion predicting DVs, controlling for dummy codes) and indirect effects were reported in standardized coefficients (β). ^†^p < 0.1, *p < 0.05, **p < 0.01, ***p < 0.001.

The alternative coding comparing the multiculturalism prime to assimilation prime also showed that these two primes had significantly different effects, through emotions, on all DVs (Table 3). *Post hoc* power analysis demonstrated a high level of power for detecting these indirect effects. When compared to the assimilation prime, the multiculturalism prime, through increasing positive emotion, significantly reduced social distancing and disgust; it also increased ethnic identity strength, HIS and AIS.

As an exploratory analysis, we also tested the specific mediating effects of the different emotions. As shown in Table 4, some but not all emotions showed significant indirect effects, suggesting avenues for future research. In particular, compared to both the control and multiculturalism primes, the assimilation prime had a strong effect on DVs through increasing anger; in contrast, no indirect effects through anger were found for the multiculturalism prime compared to control. Instead, when the multiculturalism prime was compared to control, indirect effects through pride showed that the multiculturalism prime significantly increased pride and thus indirectly affected all DVs in the direction of improved intergroup attitudes; curiously, the effects through pride were not significant in comparison to the assimilation prime, suggesting that both multiculturalism and assimilation primes evoked pride, but that the meaning of pride was different, having different effects depending on the priming context. We return to this below.

**TABLE 4:** Summary of the indirect priming effects through specific emotions in study 2.

Emotion	Indirect Effects on DV				
	Social Distancing	Disgust	Ethnic Identity	HIS	AIS
**Multiculturalism (1) vs. Control (0)**					
Proud	−0.067* [−0.128, −0.006]	−0.078* [−0.14, −0.017]	0.121** [0.035,0.206]	0.088* [0.018,0.157]	0.064* [0.01,0.119]
Interested	−0.053^†^ [−0.107,0.001]	−0.037^†^ [−0.078,0.005]	0.075* [0.001,0.149]	0.083* [0.007,0.159]	0.075* [0.006,0.143]
Happy	−0.043^†^ [−0.093,0.006]	−0.041^†^ [−0.087,0.006]	0.086* [0.007,0.166]	0.071* [0.001,0.142]	0.07* [0.001,0.139]
**Assimilation (1) vs. Control (0)**					
Anger	0.084* [0.007,0.161]	0.129** [0.036,0.221]	−0.109** [−0.19, −0.027]	−0.105** [−0.184, −0.026]	−0.095* [−0.177, −0.013]
Contempt	0.089* [0.014,0.164]	0.122** [0.032,0.212]	−0.136** [−0.221, −0.051]	−0.119** [−0.201, −0.037]	−0.114** [−0.199, −0.029]
**Multiculturalism (1) vs. Assimilation (0)**					
Interested	−0.073* [−0.13, −0.016]	−0.051* [−0.1, −0.001]	0.104** [0.027,0.181]	0.114** [0.034,0.195]	0.103** [0.031,0.175]
Happy	−0.042^†^ [−0.09,0.007]	−0.039^†^ [−0.085,0.007]	0.083* [0.003,0.163]	0.069^†^ [−0.002,0.139]	0.067^†^ [−0.001,0.136]
Anger	−0.085* [−0.162, −0.008]	−0.13** [−0.213, −0.048]	0.111** [0.032,0.189]	0.107** [0.029,0.184]	0.096* [0.016,0.176]
Contempt	−0.069* [−0.134, −0.005]	−0.095* [−0.172, −0.018]	0.106** [0.028,0.184]	0.093* [0.017,0.168]	0.089* [0.013,0.165]

Of all specific emotions, only those with significant indirect effects were reported here; no indirect effect was observed for amused, embarrassed, scared, nervous, or excited. The multicategorical dummy codes of priming and control conditions were indicated in the brackets. ^†^p < 0.1, *p < 0.05, **p < 0.01.

## Discussion

The current studies examined how Hong Kong residents react to assimilatory vs. multicultural cultural models in terms of their affective responses, social distance, attitudes toward mixed cultural symbols, and cultural identities. These findings facilitate the understanding of intergroup relations in a regional-integration cultural setting and raise a novel finding that minorities who are primed with multiculturalism over assimilation can exhibit more positive outgroup evaluations, mediated by their affective reaction to the primes.

In these studies of Hong Kong local university students, describing China as multicultural was found to increase positive emotions and therefore reduce prejudice against Mainland Chinese and strengthen Chinese identities; describing Hong Kong as assimilating into China, on the contrary, was found to decrease positive emotions and therefore worsen intergroup attitudes and weaken Chinese identities. Specifically, in Study 1, multiculturalism-priming (vs. control) among Hong Kong residents decreased social distancing toward Mainland Chinese, and although not significant, slightly lowered disgust toward Hong Kong-Mainland culture mixing and slightly increased national identity strength. But these small effect sizes suggested some complexity or counteracting effects on reactions to the multicultural-China prime. By measuring emotional reactions as a mediator and comparing the multiculturalism prime to both an assimilation prime and control, Study 2 found distinct indirect effects of these two diversity model primes: a constructive effect of multiculturalism priming– which decreased social distancing and disgust, increased ethnic identity strength, and elevated multicultural identity styles, through promoting positive emotions– and an aversive effect of assimilation priming, which increased social distancing and disgust, reduced ethnic identity strength, and suppressed multicultural identity styles, through decreasing positive emotions.

These results are mainly consistent with an interpretation that perceiving integration into China as assimilation can lead Hong Kong university students to react negatively, and emphasizes the importance of their affective responses to assimilatory vs. multicultural cultural models. They also show that more positive emotional reactions to multicultural, vs. assimilatory, perceptions of Hong Kong vis-à-vis China are linked to more positive attitudes toward Mainland Chinese, who are conversely minorities within Hong Kong. Together, the phenomena resulting from the distinct priming effects have important implications for how different perceptions of Hong Kong-Mainland integration can affect attitudes and interpersonal interactions in contemporary Hong Kong.

### Social Distancing

The effects of multiculturalism priming on prejudice toward outgroup members points to some dissimilarities between our Hong Kong participants and other studies of immigrant cultural minorities. In our studies, depictions of China as having a diverse culture - multiculturalism - was found to be beneficial for reducing social distancing from Mainland Chinese, especially in comparison to the assimilation prime. This is dissimilar from previous research among Turkish minorities in the Netherlands, where priming multiculturalism tended to decrease outgroup evaluations relative to ingroup evaluations (Verkuyten, 2005). In fact, our Hong Kong participants reacted more similarly to Dutch majority members, who were more likely to have positive evaluations of outgroup minority members after multiculturalism (vs. assimilation) primes. This reminds us that Hong Kong local residents have a relatively unique position vis-à-vis China in terms of being minority or majority members. Most encounters with Mainland Chinese may be in the context of Hong Kong, where Mainland Chinese are minorities and Hong Kong residents are majority group members. Still, the aversive reactions to the assimilation prime suggested a protective response to a perceived threat (Morrison et al., 2010; Verkuyten, 2010). This suggests that more research is needed on intergroup relations in the relatively unique contexts of regional integration, and where participants’ cultural identity may be both a minority and majority group.

### Disgust Toward Regional Culture Mixing and Chinese Identities

A belief that the local culture is a valuable part of a broader entity could in fact be perceived as beneficial to the ingroup, thus encouraging an interest in cohesion of Hong Kong and Mainland China. Contrasting effects between multicultural and assimilation ideologies were observed in regard to the perceived compatibility of the two cultures, mediated by emotional reactions. It is likely that to the degree that multiculturalism reduced Hongkongers’ perceived sense of threat, it enhanced the perceived compatibility of these cultures, as shown by reduced disgust toward mixed cultural symbols and increased integration of Chinese and Hong Kong identities; while assimilation had the opposite effects. This process revealed how these two diversity models affected individual’s alienation vs. integration of two cultures.

It is worth comparing our results with the recent Cheon and Hong (2020) study, in which in general, patriotism toward Hong Kong was associated with more disgust toward images associated with Mainland China. Cheon and Hong (2020) also found that their Hong Kong participants evidenced stronger aversive reactions to cultural symbols involving Mainland Chinese culture, compared to those involving American symbols. In both studies here, we found that disgust was positively correlated with social distancing from Mainland Chinese, and that it was similarly increased by the assimilation prime, suggesting that disgust indexed attitudes toward outgroups in a way that went beyond a simple aversive reaction to culture mixing. Findings add to this evidence that disgust toward cultural mixing is associated with prejudice and rejection of the “other” culture. Moreover, our Study 2’s findings that effects were mediated through emotions suggest that the effects of ingroup identity on disgust may depend on participants’ interpretation; especially, whether or not participants perceive a sense of threat (Morrison et al., 2010). In our study, the negative emotion reactions may be the product of perceived cultural or identity threat posed by the assimilation prime, a more specific mediator which should be investigated in future studies.

In Study 2, it was noted that the indirect priming effects on hybrid (HIS) and alternating identity styles (AIS) were similar, though these are different identity management strategies that in past research have been associated with different adaptation outcomes. Previous work has shown that HIS is associated with identity consolidation, in contrast to AIS which is associated with identity conflict (Ward et al., 2018). However, as outcome variables in this study, both HIS and AIS appear to index forms of accepting a Chinese identity, leading them to perform similarly to one another and to the measures of an overall ethnic Chinese identity. It remains to be seen how HIS and AIS approaches to Hong Kong-Chinese identities function distinctively as predictors of well-being and other adaptation outcomes in the context of Hong Kong.

### Emotional Reactions to Diversity Models

The importance of emotion as an explanation of different reactions to diversity models deserves further research. The results are an interesting complement to findings of Morrison et al. (2010), who found that White Americans’ negative reaction to multiculturalism primes (vs. color-blindness) was mediated by their sense of symbolic threat; here, we find that Hong Kong participants’ negative reaction to an assimilation prime (vs. multiculturalism) was mediated by more negative emotional reactions.

By additionally exploring mediating effects of specific emotions, we observed sensible effects that suggest some emotions may be more important than others in understanding how diversity models affect intergroup relations. Here, the exploratory analyses suggested that the positive effects of multiculturalism priming went through increasing pride, happiness and feeling interested, while the negative effects of assimilation were observed through its increase of anger and contempt. These findings were consistent with the important role of emotions in mediating the effect of intergroup contact on outgroup prejudice (Mackie and Smith, 2015). As a strong signal of intergroup attitude, the specific effects of assimilation on anger and contempt demonstrated in the current study was informative regarding the intergroup relations in Hong Kong. While anger is more commonly experienced in western cultures (Kitayama et al., 2006; Kimel et al., 2017), both anger and contempt are “distancing” emotions; in the context of intergroup relations, they may be especially indicative of a desire to delineate group boundaries.

In past research, the effect of positive intergroup emotions has been understudied. Pride, for example, has had inconsistent associations with intergroup attitudes depending on context; increased endorsement of universalism values has been associated with stronger national pride (Du et al., 2019), but, among Western (though not non-Western) cultures, stronger national pride was negatively associated with endorsing diversity (Hamamura, 2017). In the present study, although pride was not evoked more by the multiculturalism vs. assimilation prime, pride had benignant effects on attitudes and identities after the multiculturalism prime but not when evoked by the assimilation prime. It is plausible that the multiculturalism prime specifically evoked pride in China, or in both Hong Kong and China, thus resulting in more positive attitudes, and suggests that pride is not necessarily a distancing or defensive emotion. Different effects of pride deserve further investigation.

### Limitations and Future Directions

The two studies were slightly underpowered due to limited sample size. Thus, small priming effects might not have been detected. In Study 1, the effect of Multiculturalism vs. Control on social distancing was significant, but this main effect in comparison to Control condition was not observed in Study 2 and its indirect effect was only marginally significant, suggesting that effects, especially of multiculturalism compared to neutral conditions, may be weak and in need of replication. In addition, the two studies also employed convenience sampling methods among Hong Kong university students, which may not generalize to all sectors of the Hong Kong population.

In these studies, we conceptualized identity measures as dependent variables and included them immediately after the experimental manipulations, precluding their use as individual-difference moderators of reactions. As ethnic identity strength has been found to be an important moderator of reactions to multicultural vs. assimilatory frames (e.g., Verkuyten, 2009; Morrison et al., 2010), this is a clear avenue for future research. This is especially relevant in the context of Hong Kong, where the degree to which one identifies as Chinese and/or Hong Konger has been associated with many aspects of attitudes toward China in past research (e.g., Hong et al., 1999; Lam et al., 1999, 2006; Hong et al., 2004). Moreover, as the two multicultural identity styles were examined as outcomes rather than predictors, the distinctions between hybrid identity style and alternating identity style that can be found through controlling for one another (Ward et al., 2018) and thus more complex features of these cultural identities could not be thoroughly examined. Further study is needed to explore the distinct functions of these two identity styles.

Caution should be advised against assuming multiculturalism priming will always have positive effects. Some studies have demonstrated a backlash against multicultural ideologies (Verkuyten and Yogeeswaran, 2019; Verkuyten et al., 2020); for example, majority individuals who had stronger ingroup identification and perceived their values to be threatened by such ideologies exhibited greater outgroup prejudice (Morrison et al., 2010). On the other hand, different ways of delivering a multiculturalism message could result in different reactions. Past research has also found that reading descriptions that solely describe the broad goals of multiculturalism can make the majority group feel less threatened by immigrants, resulting in less prejudice, but reading the concrete details of how to implement multiculturalism had the opposite effects (Mahfud et al., 2018). To encourage positive effects, presentations of diversity models should be tailored to the context and circumstances.

### Summary

The current study employed an experimental design that demonstrated the positive influence of multiculturalism priming on reducing social distancing, disgust toward Hong Kong-Mainland culture mixing, and on strengthening the endorsement of a superordinate ethnic Chinese identity and perceived compatibility of Hong Kong and Chinese identities among Hong Kong local university students. It also demonstrated a salient aversive effect of assimilation priming. Understanding the changing relations in contemporary Hong Kong, caution is advised regarding considerations of how public messages about integration with China should be delivered and attention paid to what emotional responses they will evoke. The distinct reactions of our Hong Kong participants to these two types of diversity models adds another piece to the puzzle of understanding intergroup emotions and intergroup relations in different contexts. For the participants in these studies, the preferable way to place local culture in the grand Chinese framework seems to be to retain its uniqueness, which at the same time increased their interest in being an integrated part of the greater diverse entity. Such unique feelings deserve attention in further investigations.

## Data Availability Statement

The datasets presented in this study can be found in online repositories. The names of the repository/repositories and accession number(s) can be found below: https://osf.io/fdxt6/.

## Ethics Statement

The studies involving human participants were reviewed and approved by The Human Research Ethics Committee of the Education University of Hong Kong. The patients/participants provided their written informed consent to participate in this study.

## Author Contributions

FY and EB conceptualized the studies. FY conducted the data collection and analyses, and wrote the draft. EB supervised all studies, reviewed, and edited the draft. Both authors contributed to the article and approved the submitted version.

## Conflict of Interest

The authors declare that the research was conducted in the absence of any commercial or financial relationships that could be construed as a potential conflict of interest.

## Publisher’s Note

All claims expressed in this article are solely those of the authors and do not necessarily represent those of their affiliated organizations, or those of the publisher, the editors and the reviewers. Any product that may be evaluated in this article, or claim that may be made by its manufacturer, is not guaranteed or endorsed by the publisher.

## Footnotes

1 Additionally, data from three participants were excluded based on Mahalanobis distance (Tabachnick and Fidell, 2007), and data from an additional two participants were also excluded due to incomplete surveys.

2 A power calculation performed in G^∗^power taking the smallest effect size value of *d* = 0.47 reported in previous studies (Cohen’s d ranged from.47 to.91; Todd and Galinsky, 2012) found that 146 participants were required to obtain 80% power for independent-group t-tests with 5% alpha (two-tailed). Given these assumptions, the sample size of 118 participants is therefore slightly underpowered. Participants were recruited from a convenience sample of Hong Kong-origin university students in Hong Kong. The data collection process continued for around three weeks, until the response rate was very low.

3 For Study 1, we conducted an independent sample t-test and found age did not differ between the two conditions, *t*(112) = 0.17, *p* = 0.87. The Chi-square test also showed no gender difference between conditions, χ2 (2) = 0.97, *p* = 0.62. Subsequent ANCOVA tests with conditions as fixed factor, age and gender as covariates demonstrated the same priming effects as discussed above. Similarly, for Study 2, an ANOVA showed that age did not differ among the three conditions, *F*(2, 157) = 11.65, *p* = 0.51. Gender also did not differ between conditions, χ2 (2) = 0.81, *p* = 0.67. The main effects of priming conditions for all models were consistent with models that did not control the covariates.

4 Participants were recruited on a university campus in Hong Kong. Participation was open to all undergraduate and graduate students except the participants of Study 1. The recruiting process continued for about two months and stopped upon the end of the semester.

5 This study was conducted as part of a larger dissertation project, with only some analyses (H1-3) preregistered (https://osf.io/dyw39). In the pre-registration, a typical social psychology effect size value (*r* = 0.21; Richard et al., 2003) was also used to calculate the required sample size prior to data collection, and 210 participants were required to achieve 80% power. Additional measures and analyses were included in the survey and pre-registration that are not relevant to the focus of this article (on how diversity models affect Hong Kong participants’ intergroup attitudes (prejudice- and identity-relevant DVs) and so are not discussed here (including a measure of Chinese/Western identity before the presentation of the primes, acculturation attitudes, and well-being measures).

6 An alternative way of analyzing the data using positive affect and negative affect as separate variables was also performed. Interested readers could refer to the Supplementary Materials.

7 Adding evidence that the null direct effect of primes on outcomes may be due to suppression factors, we also observed that direct effects had opposite signs to the indirect effects. For example, controlling for positive emotion, the unstandardized regression coefficient of multiculturalism prime (contrasting to assimilation prime) predicting social distancing was positive though not significant (*B* = 0.18, *p* = 0.49); in the meantime, the indirect effect of multiculturalism prime through positive emotion was negative (*B* = −0.44, *p* < 0.001). As such, these two opposite effects might result in a non-zero but a non-significant total effect (MacKinnon et al., 2000).
