# Supplementary material for: Multiculturalism, Culture Mixing, and Prejudice: Effects of Priming Chinese Diversity Models Among Hong Kong University Students
Source: Front Psychol. 2021 Jul 23;12:691858. doi: 10.3389/fpsyg.2021.691858 (PMC8343399; doi:10.3389/fpsyg.2021.691858)
Supplement: Supplementary file 1 [file Table_1.DOCX]

**Multiculturalism, Culture Mixing, and Prejudice: Effects of Priming Chinese Diversity Models among Hong Kong University Students - Supplementary Material**

1. **Study 2, Additional Analyses with Positive Affect and Negative Affect Separately**
2. **Sample images used for Disgust towards Culture Mixing**

**(1) Study 2, Additional Analyses with Positive Affect and Negative Affect Separately**

In the main text, for the sake of brevity and clarity, we combined all PANAS items into a single measure of positive minus negative affect items, i.e. a single measure of “positive emotion.” Here, we show the same analyses when using positive affect and negative affect items as separate scales of “positive affect” and “negative affect.” Conclusions are unchanged. It may be of interest to note that indirect effects of the multiculturalism prime were mediated through positive affect, while indirect effects of assimilation priming were mediated through negative affect.

***Main Effect of Priming Conditions on Positive and Negative Affect Separately***

A multivariate analysis of variance (MANOVA) was conducted with priming conditions as fixed factors and separate scales of positive affect and negative affect as dependent variables; results suggested a significant main effect of priming conditions on the dependent variables, *F*(2,165) = 9.68, *p <* .001, Wilk’s *λ* = .80. Separate ANOVA tests indicated that the effect of priming conditions was significant on positive affect, *F*(2,165) = 4.95, *p <* .05, *η*^2^ =.05. Post hoc tests with Holm correction showed that the multiculturalism prime had a higher positive affect score than both the control condition, *t =* 2.46, *p <* .05, Cohen’s *d =* .46; *M_M_* = 2.78, *SD* = 1.22; *M_control_* = 2.27, *SD* = 1.01, and assimilation prime, *t =* 2.40, *p <* .05, Cohen’s *d =* .43; *M_A_* = 2.28, *SD* = 1.11. No significant difference was observed between the assimilation and control conditions, *t =* -.04, *p =* .97, Cohen’s *d =* -.01.

The effect of priming conditions was also significant on negative affect, *F*(2,165) = 12.76, *p <* .001, *η*^2^ =.13. Post hoc tests with Holm correction showed the assimilation condition had a higher negative affect score than both the control condition, *t =* 4.52, *p <* .001, Cohen’s *d =* .81; *M_A_* = 2.44, *SD* = 1.23; *M_control_* = 1.58, *SD* = .85, and multiculturalism condition, *t =* 4.26, *p <* .001, Cohen’s *d =* .76; *M_M_* = 1.64, *SD* = .86. No significant difference was observed between the multiculturalism and control conditions, *t =* .30, *p =* .76, Cohen’s *d =* .07.

Thus, consistent with and in addition to the evidence described in the main text that multiculturalism increased positive emotions and assimilation decreased positive emotions, the separate analyses suggested that the multiculturalism prime primarily promoted positive affect while the assimilation prime primarily increased negative affect.

***Indirect Effects of Priming Conditions on Outcomes through Positive and Negative Affect Separately***

In addition, we also tested the mediating effects of priming conditions on social distancing, disgust, ethnic identity strength and HIS and AIS through positive affect and negative affect separately. The hypothesized path model (Figure 1) was tested using lavaan (Rosseel, 2012) and joint significance of indirect paths were estimated by maximum likelihood estimation with robust standard errors.


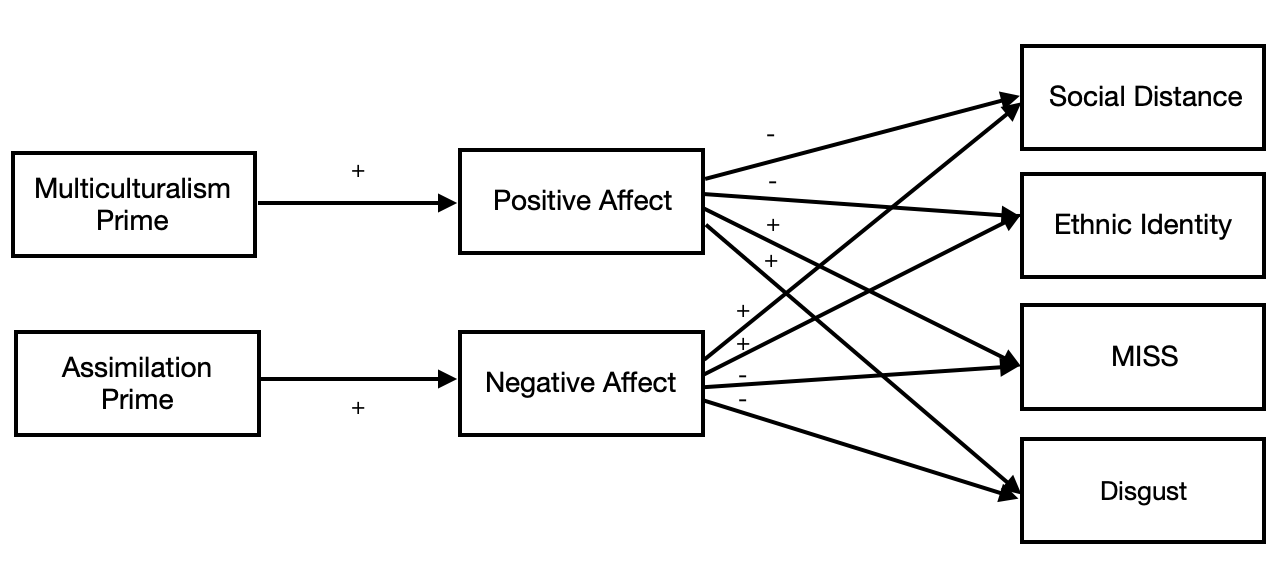


*Figure 1.* Hypothesized path model showing the effects of the Multiculturalism prime and Assimilation prime (each compared to Control) on ethnic identity strength, social distancing, multicultural identity style (MISS; operationalized as AIS and HIS separately), and disgust, mediated by Positive and Negative Affect.

Results demonstrated an excellent overall model fit, *χ2* = .11, *df* = 2, *p* = .95, CFI = 1.0, TLI = 1.06, RMSEA = .00 [.00, .06], *p_close-fit_* = .94, SRMR = .003. The joint significance of mediating paths was also tested.

The multiculturalism prime reduced social distancing through positive affect, *β* = -.07, *p* < .05, 95% CI [-.12, -.01]; the assimilation prime increased social distancing through negative affect, *β* = .08, *p* < .05, 95% CI [.01, .15].

The multiculturalism prime reduced disgust through positive affect, *β* = -.07, *p* < .05, 95% CI [-.12, -.01]; the assimilation prime increased disgust through negative affect, *β* = .12, *p* < .001, 95% CI [.05, .19];

The multiculturalism prime increased ethnic identity strength through positive affect, *β* = .12, *p* < .01, 95% CI [.03, .21]; the assimilation prime reduced ethnic identity strength through negative affect, *β* = -.12, *p* < .001, 95% CI [-.19, -.06];

The multiculturalism prime, through positive affect, increased HIS, *β* = .10, *p* < .01, 95% CI [.03, .17], and AIS, *β* = .09, *p* < .01, 95% CI [.03, .16]; while the assimilation prime, through negative affect, decreased HIS, *β* = -.11, *p* < .01, 95% CI [-.18, -.05], and AIS, *β* = -.09, *p* < .05, 95% CI [-.16, -.02].

The above results showed indirect effects of the multiculturalism prime being mediated through positive affect, and indirect effects of assimilation priming being mediated through negative affect, on ethnic identity strength, MISS, social distancing and disgust towards culture mixing.

**(2) Sample images used in the current studies.**

| Group | Ingroup | Outgroup | Mixed |
| --- | --- | --- | --- |
| Hong Kong | 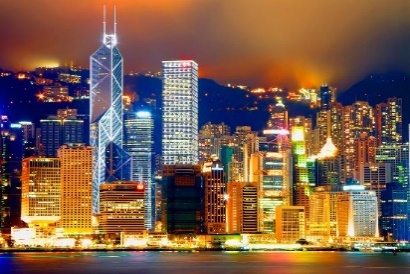 | 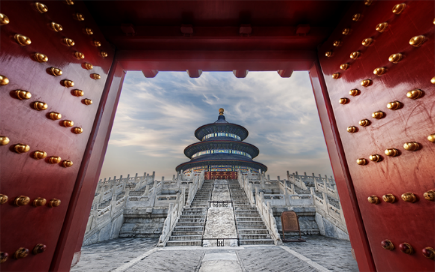 | 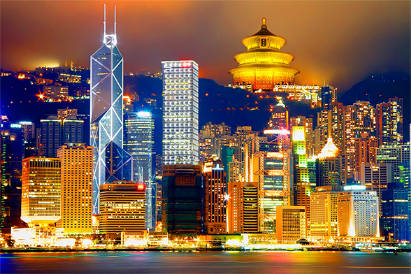 |
| Mainland | 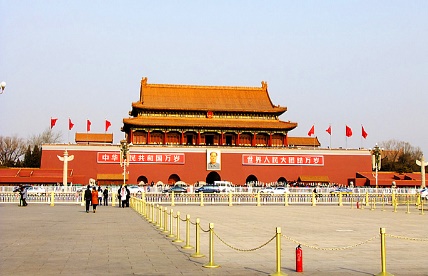 | 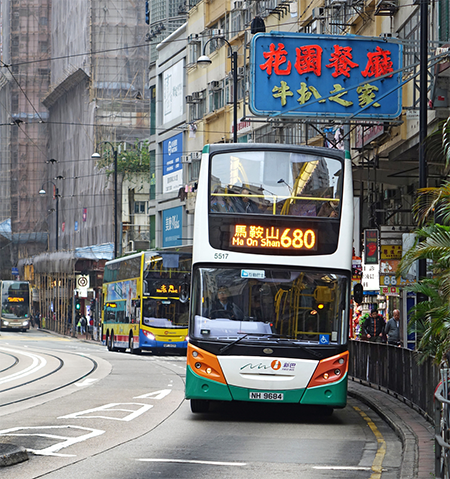 | 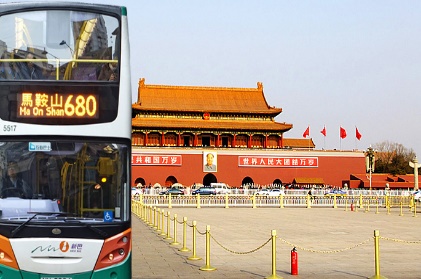 |
